# Data on electrical energy conservation using high efficiency motors for the confidence bounds using statistical techniques

**DOI:** 10.1016/j.dib.2016.06.004

**Published:** 2016-06-14

**Authors:** Muhammad Mujtaba Shaikh, Abdul Jabbar Memon, Manzoor Hussain

**Affiliations:** a Department of Basic Sciences and Related Studies, Mehran University of Engineering and Technology, Jamshoro, Pakistan; b Department of Electrical Engineering, Mehran University of Engineering and Technology, Jamshoro, Pakistan; c Government of Pakistan

## Abstract

In this article, we describe details of the data used in the research paper “Confidence bounds for energy conservation in electric motors: An economical solution using statistical techniques” [1]. The data presented in this paper is intended to show benefits of high efficiency electric motors over the standard efficiency motors of similar rating in the industrial sector of Pakistan. We explain how the data was collected and then processed by means of formulas to show cost effectiveness of energy efficient motors in terms of three important parameters: annual energy saving, cost saving and payback periods. This data can be further used to construct confidence bounds for the parameters using statistical techniques as described in [1].

**Specifications Table**

**Table:** 

Subject area	Energy economics, statistical analysis
More specific subject area	Electrical energy conservation using energy efficient motors
Type of data	Tables, figures and graphs
How data was acquired	Using motor data and energy consumption forms while personal visits at surveyed industries
Data format	Raw, filtered, analyzed
Experimental factors	Factors needed to calculate energy saving, cost saving and payback period by a similar rating energy efficient motor in place of an existing standard efficiency motor
Experimental features	Statistical analysis of the data using student׳s *t*-distribution in terms of confidence bounds of the energy conservation parameters.
Data source location	1. Hyderabad, Pakistan ( latitude 25.367°N, longitude 68.367°E)
	2. Jamshoro, Pakistan (latitude 25.26°N, longitude 68.20°E)
Data accessibility	Data is available with this article

**Value of the data**

• The data highlights the benefits of using high efficiency motors in place of existing standard efficiency motors in industrial sector of Pakistan in terms of annual energy savings, cost savings and payback periods.
• The annual energy consumption and annual operational cost are compared for a sample data of 20 standard and energy efficient motors (EEMs).
• The data described in this article supports the step by step statistical analysis in terms of confidence bounds for the three parameters: annual energy savings, cost savings and payback periods for EEMs.
• The confidence bounds based on the discussed data which are presented in associated research paper [1] can be used to attract financers for large scale purchase and replacement of standard motors (SMs) by EEMs to conserve sufficient amount of electrical energy.
• The procedure described in this article and the underlying research paper [1] can be used by researchers for processing the data related to high efficient motors in other countries (the data in Refs. [2], [3], [4], [5], [6], [7], [8], [9], [10]) in order to generate encouraging confidence bounds to promote electrical energy conservation using EEMs [1].

## 1 Data

The data in this work describe general information about existing low-efficiency motors at surveyed sites, like: motor application type, utility rate being used, annual operating hours and related information from motor nameplates. We also present the comprehensive values of some important parameters for each standard motor in the sample like: input volts, input amperes, input kW, operating speed (in rpm) and power factor.

## 2 Experimental design, materials and methods

**Fig. 1:**
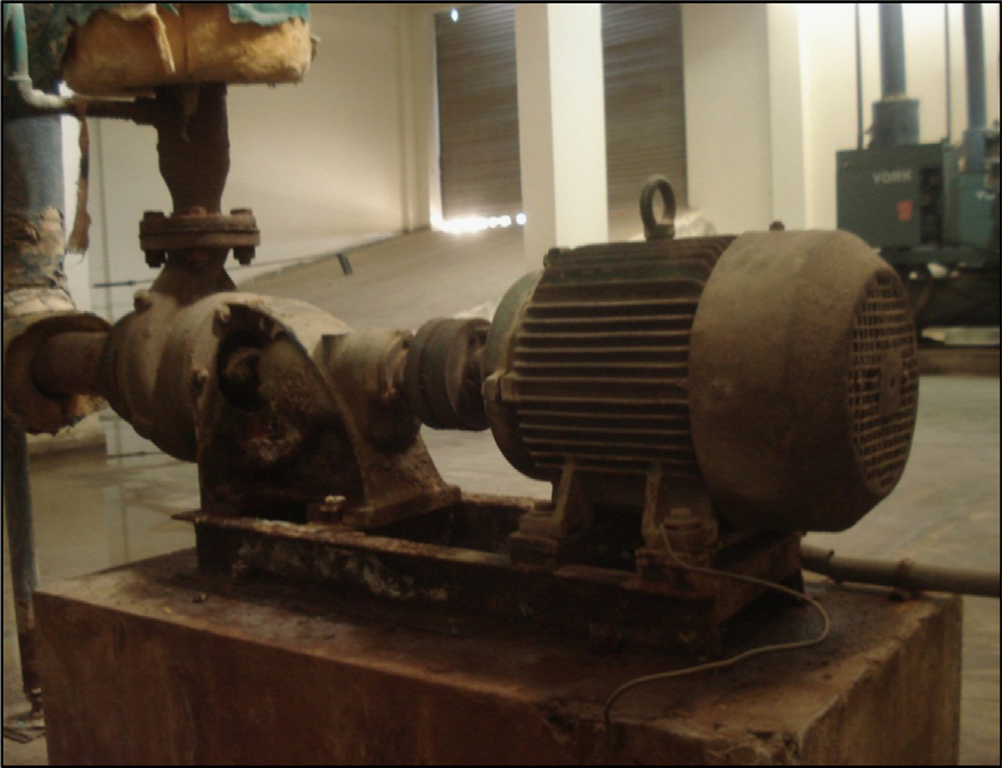
2.1 HP motor used for dearator transfer pump.

**Fig. 2:**
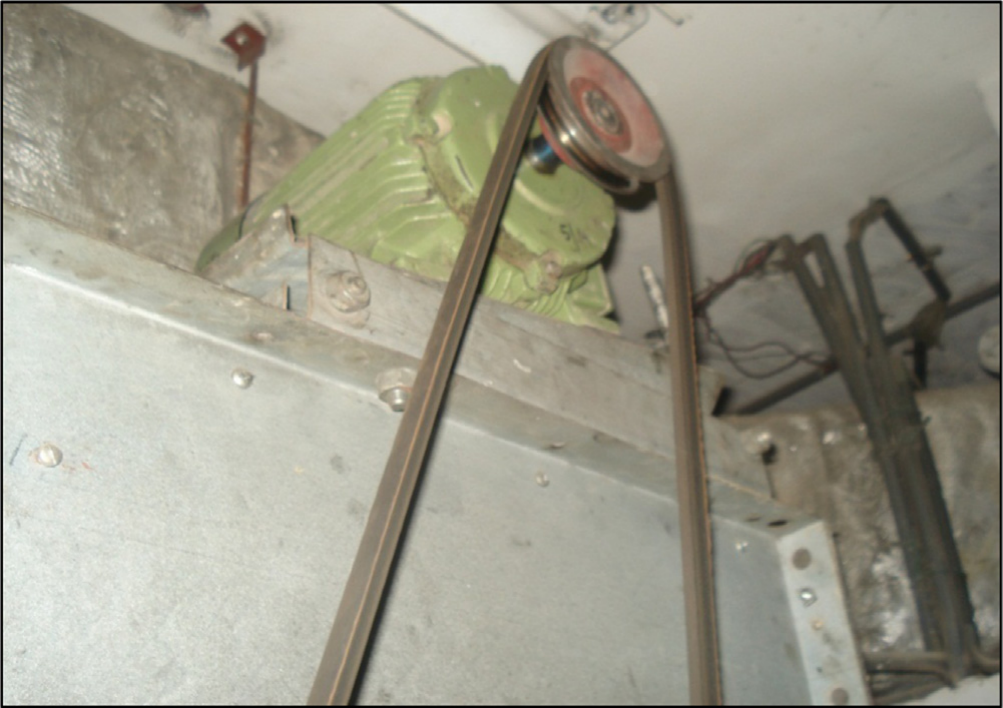
2.2 kW motor used for AC chiller plant.

**Fig. 3:**
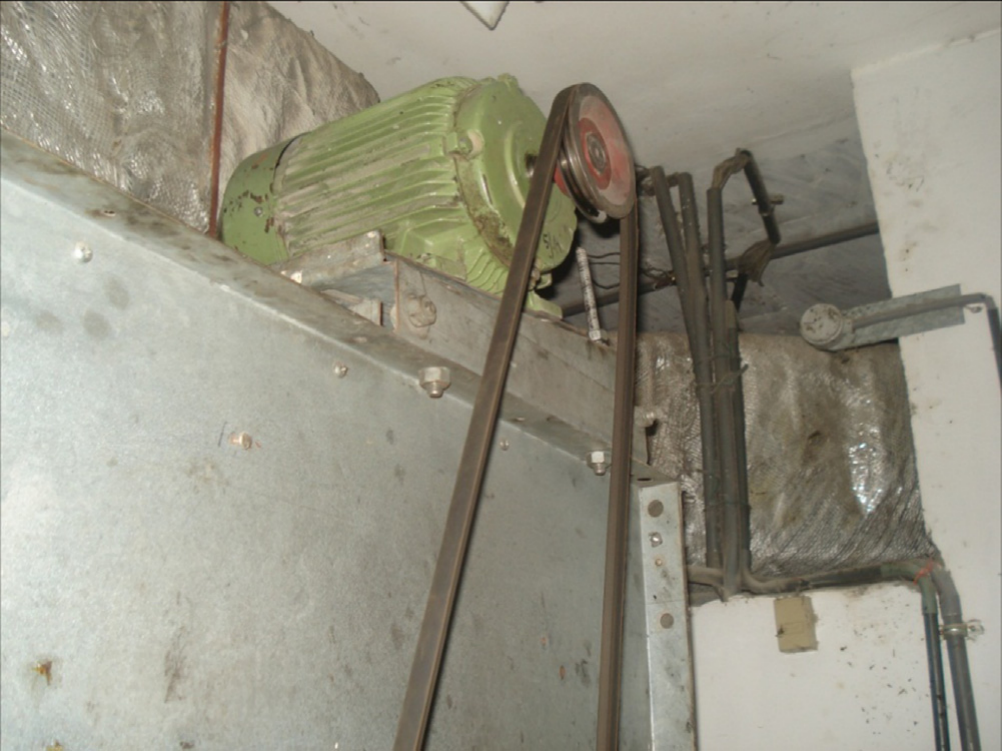
1.6 HP motor used for water treatment.

**Fig. 4:**
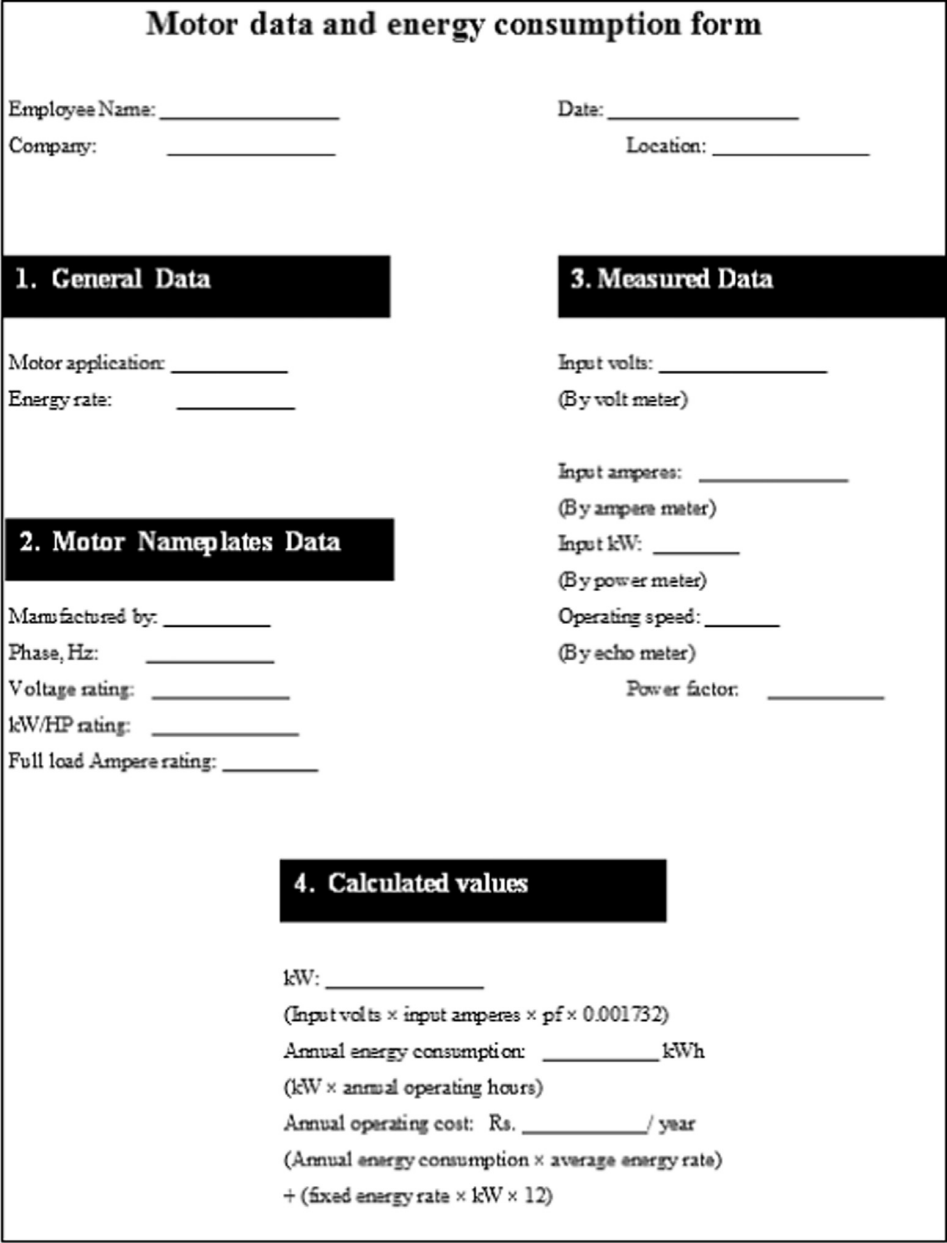
Motor data and energy consumption form.

The data presented in this article is basis for the statistical analysis in terms of confidence bounds – as discussed in [1] – for the annual energy savings, cost savings and payback periods of EEMs when replaced for SMs. To acquire the necessary data, some industries based at Pakistan were surveyed and data on existing standard efficiency motors were noted while personal visits. We describe in next section, the acquired data, its processing using different formulas and its descriptive statistical analysis which is required for the construction of confidence bounds. To gather the best possible sample of existing standard efficiency motors, we surveyed the industries where majority of the motors were installed. Surveyed industries included: Pakistan Steel Mill (PSM), Karachi; Thermal Power Station, Jamshoro; Regional Control Centre, Jamshoro; and, Water Works/Pumps, Hyderabad. The data was obtained in the year 2011 while personal visits to the surveyed industries. Some photos of motors at survey sites are given in Fig. 1, Fig. 2, Fig. 3 according to their applications. The important electrical parameters related to the installed standard efficiency motors were noted on the *motor data and energy consumption form,* a sample in Fig. 4. For reference, a completely filled form for 2HP motor can be found as Fig. 1 of [1]. The comprehensive data measured and noted in these forms for all 20 sample SMs is gathered in Table 1. The values in Table 1 can be used to calculate power (in kW), annual energy consumption (in kWh/year) and annual operational cost (in Rs./year) for the 20 sample SMs.

Similar rating EEMs with price and efficiency detail comparison with those of SMs (as per Table 1 in [1]), when processed with the cost effectiveness formulas and conditions given in [1], resulted encouraging values of annual energy savings, cost savings and payback periods. An example of such calculation is given here to support the statistics in the main paper, i.e. Ref. [1].

## 3 Example calculation of cost effectiveness parameters for a 2HP motor

The following analysis for 2HP motor operating at 75% of full rated load illustrates how to determine the cost effectiveness of obtaining an energy-efficient versus a standard efficiency motor for the initial purchase case. The formulas are used from Ref. [11].

• **Kilowatts saved:**
  (1) $$\mathrm{KW}\;Saved\;\;=\;\text{HP}\times L\times 0.746\left[\frac{100}{\xi_{\mathrm{SM}}}\;-\;\frac{100}{\xi_{\mathrm{EM}}}\right]=\;\text{2}\times 0.75\times 0.746\left[\frac{100}{79.8}\;-\;\frac{100}{83.8}\right]=\;0.066933$$
  This is the amount of energy conserved by the energy efficient motor during each hour of use.
  Annual energy savings are obtained by multiplying by the number of operating hours at the indicated load.
• **Energy saved:**
  (2) $$\mathrm{kWh}\;\text{Savings}\;=\mathrm{Operating}\;\mathrm{hours}\;{\times P}_{\mathrm{input}}\;\mathrm{Saved}=8640\times 0\text{.066933}\;=\;\text{578}.305$$
• **Annual cost savings:**
  (3) TotalCostSavings=(kWSaved×12×MonthlyDemandCharge)+(kWhSavings×EnergyCharge)=(0.066933×12×380)+(578.305×9)=Rs.5509.96
  In this example, installing an energy-efficient motor reduces utility billing by Rs. 5509.96 per year. The simple payback for the incremental cost associated with an energy efficient motor purchase is the ratio of discounted list price premium (see Table 1 in [1]) or incremental cost to the total annual cost savings. A list price discount of 75% is used in this analysis.
• **Payback period**

(4) $$\mathrm{Simple}\;\text{Payback}\;=\frac{\mathrm{List}\;\mathrm{Price}\;\text{Premium}\;\times \;\text{Discount}\;\mathrm{Factor}}{\mathrm{Total}\;\mathrm{Annual}\;\mathrm{Cost}\;\mathrm{Savings}}=\;\frac{1650\times 0.75}{5509.96}=0.22459$$

Thus, the additional investment required to buy this energy efficient motor would be recovered within 0.22459 years.

**Fig. 5:**
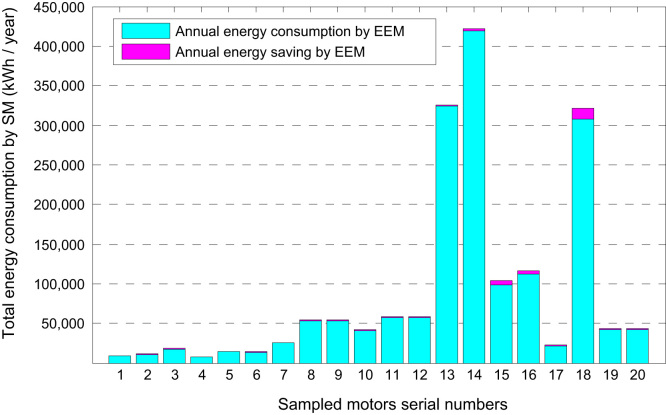
Comparison of total annual energy consumption by 20 SMs and EEMs.

**Fig. 6:**
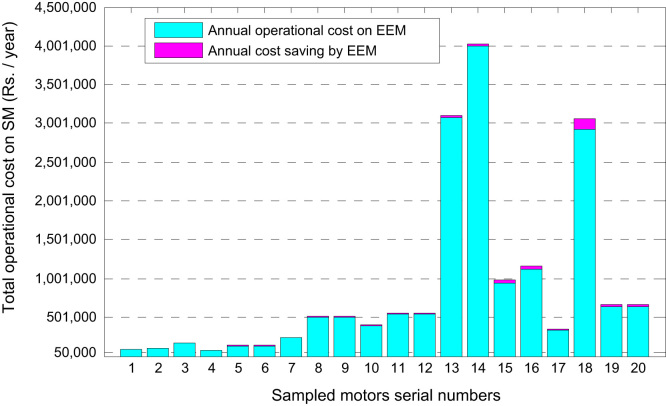
Comparison of annual operational cost on 20 SMs and EEMs.

Similar analysis for other motors in the sample lead to the 20 values for each parameter: annual energy saving, cost saving and payback periods for replaced EEMs for SMs. Fig. 5 shows the break-up of total energy consumption by sampled SMs in terms of energy consumption by similar rating EEMs and corresponding energy savings. Fig. 6 describes that the sum of annual operational cost on an EEM and its corresponding cost savings equals the annual operational cost on similar rating SM. Finally, the data described in this article is further analyzed statistically in Ref. [1] to construct the confidence bounds for energy conservation parameters.

## Figures and Tables

**Table 1:** Summary of measured electrical parameters for 20 sample SMs.

**Serial number**	**Motor rating (HP)**	**Motor rating (kW)**	**Measured voltage (V)**	**Measured current (A)**	**Measured power (kW)**	**Power factor**	**Annual operating (h)**
**1**	1.5	1.119	355	2	1.11	0.90	8640
**2**	1.6	1.1936	355	2.4	1.301	0.88	8640
**3**	2	1.492	370	3.7	2.133	0.90	8640
**4**	2.0107	1.5	354	2.9	1.77	0.96	4320
**5**	2.1	1.5666	354	2.9	1.77	0.96	8640
**6**	2.9490	2.2	369	5.2	3.19	0.96	4320
**7**	3	2.238	369	5.2	2.99	0.90	8640
**8**	4	2.984	210	19	6.21	0.90	8640
**9**	4.6	3.4316	210	19	6.21	0.90	8640
**10**	5	3.73	373	8.4	4.88	0.90	8640
**11**	7.3726	5.5	380	11	6.8	0.94	8640
**12**	7.5	5.595	380	11	6.8	0.94	8640
**13**	10	7.46	419	65	37.73	0.80	8640
**14**	10.0536	7.5	420	84	48.56	0.80	8640
**15**	20.10724	15	420	19	12.02	0.87	8640
**16**	40.21448	30	410	43	27.42	0.88	4320
**17**	49.59786	37	418	54	41.701	0.80	720
**18**	73.72654	55	419	64	39.94	0.80	8640
**19**	80.42895	60	403	108	60.255	0.80	720
**20**	100.5362	75	416	105	61.501	0.80	720
