# Supplementary material for: Data on electrical energy conservation using high efficiency motors for the confidence bounds using statistical techniques
Source: Data Brief. 2016 Jun 14;8:529–35. doi: 10.1016/j.dib.2016.06.004 (PMC4925456; doi:10.1016/j.dib.2016.06.004)
Supplement: Supplementary file 1 — Supplementary material [file mmc1.docx]

**CONFLICT OF INTEREST STATEMENT**

**DIB-D-16-00374**

**“**Data on electrical energy conservation using high efficiency motors for the confidence bounds using statistical techniques**”.**

We, authors of this data article, state that we don’t have any conflict of interest with any researcher, editorial member, reviewer, organization, etc.

Regards,

Muhammad Mujtaba Shaikh (corresponding author)

Email: [mujtaba.shaikh@faculty.muet.edu.pk](mailto:mujtaba.shaikh@faculty.muet.edu.pk)

Cell # +92-333-2617602
